# Association among physical activity, stress and oral health among university students: A cross-sectional study

**DOI:** 10.1097/MD.0000000000050759

**Published:** 2026-09-18

**Authors:** Qianru Li, Zhe Chen, Chenyuan Sun, Shengyun Lin, Xinhan Yang, Jian Li

**Affiliations:** a Department of Stomatology, Xiang’an Hospital of Xiamen University, School of Medicine, Xiamen University, Xiamen, China; b Department of Oromaxillofacial Head and Neck Surgery, Center for Oromaxillofacial Head and Neck Disorders, Huashan Hospital, Fudan University, Shanghai, China; c Shanghai Engineering Research Center of Tooth Restoration and Regeneration & Tongji Research Institute of Stomatology & Department of Implantology, Shanghai Tongji Stomatological Hospital and Dental School, Tongji University, Shanghai, China.

**Keywords:** oral health, physical activity, stress, university students

## Abstract

This study aims to comprehensively examine the interrelationships among physical activity, stress, and oral health status in university students to provide empirical evidence for targeted oral health promotion strategies. A survey was conducted among current university students in mainland China. Physical activity was evaluated via the International Physical Activity Questionnaire Short Form, and stress was measured through the 14-item Perceived Stress Scale. Oral health status was evaluated through self-reported indicators, including gingival bleeding, toothache, and oral ulcer. Data were analyzed using multivariable logistic regression and structural equation modeling. A *P* value < .05 was considered statistically significant. A total of 1578 participants were included. Findings revealed that 47.7% of students had insufficient physical activity, while 38.7% reported high or excessive stress. Multivariable logistic regression showed that insufficient physical activity and elevated stress levels were associated with an increased risk of gingival bleeding and oral ulcers (*P* < .05). Structural equation modeling showed that physical activity was negatively associated with perceived stress (standardized coefficient = −0.360, *P* < .05), and perceived stress was positively associated with self‑reported oral symptoms (standardized coefficient = 0.411, *P* < .05). However, the direct association between physical activity and oral symptoms was not statistically significant (standardized coefficient = −0.009, *P* = .765). Physical activity, stress, and oral health are closely intertwined. Insufficient activity and high stress are critical determinants of poor oral health among university students. The observed association between physical activity and oral symptoms was mediated primarily through perceived stress rather than a direct effect. Universities should implement integrated interventions that encourage regular exercise and provide mental health support to enhance the holistic and oral well-being of the student population.

## 1. Introduction

Oral health encompasses the capacity to smile, taste, speak, smell, chew, swallow, and experience oral sensory input, as well as to convey emotions via facial expressions, in a confident manner and free from pain, discomfort, or pathological conditions involving the craniofacial complex.^[1]^ It is a fundamental component of general health and is closely linked to social, physical, psychological, and economic well‑being.^[2]^ Oral diseases are highly prevalent across the world and impose a substantial economic burden. A previous report estimated that approximately 3.47 billion people are affected by oral conditions.^[3]^ In 2010, global dental care expenditures for these conditions were projected at 298 billion US dollars, accounting for 4.6% of total health‑care spending.^[4]^ More recently, the World Economic Forum reported that poor oral health results in approximately 710 billion US dollars annually in treatment costs and productivity losses.^[5]^ Improving oral health at the population level is therefore expected to yield substantial gains in overall well‑being. Identifying the determinants that influence oral health is essential for designing and implementing effective oral health management strategies.

Physical activity describes all types of physical movements that rely on skeletal muscles and result in higher energy output and encompasses sports, occupational tasks, household chores, exercise training, and routine daily activities.^[6]^ To promote and maintain health-related fitness, the World Health Organization recommends engaging in a minimum of 150 minutes per week of moderate‑intensity physical activity or 75 minutes per week of vigorous‑intensity activity.^[7]^ Multiple studies have explored the impact of physical activity on oral health outcomes. In 1 cohort of 2521 adults, individuals with higher activity levels exhibited a reduced prevalence of periodontitis.^[8]^ Similarly, physically active adolescents demonstrate more favorable oral health behaviors than their less active peers.^[9]^ In addition, a recent study of Japanese women showed a linear, inverse association between physical activity and both the occurrence and severity of periodontal disease.^[10]^ These findings may be partly attributable to regular physical activity downregulating systemic inflammatory mediators.^[11]^ Collectively, these data indicate that physical activity may confer beneficial effects in the prevention and management of oral diseases.

Oral health is closely intertwined with both general health status and psychological stress. Within a biopsychosocial framework, perceived stress is a key psychological construct that can shape health-related behaviors, including smoking, which is frequently adopted as a maladaptive coping strategy.^[12]^ Persistent stress may disrupt multiple physiological systems and has been implicated in the onset and progression of periodontal disease, recurrent oral ulcerations, burning mouth symptoms, and xerostomia.^[13]^ Higher self-reported stress has been linked to higher DMFT indices. Research conducted by Ben Hassan and colleagues further demonstrated a notable positive correlation between stress status and DMFT among medical undergraduates.^[14]^ Furthermore, stress has been linked to poorer self-rated oral health and reduced quality of life.^[15]^ Collectively, these observations support the inclusion of perceived stress, alongside physical activity and oral health, within a unified analytical model to investigate both direct effects and potential mediating pathways.

Building on the above evidence, it is important to examine physical activity, perceived stress, and oral health among university students, who are in a critical transitional period and may be vulnerable to both unhealthy behaviors and psychological distress. Although university students often experience substantial academic and social pressures, evidence regarding how physical activity and perceived stress interact to influence their oral health remains limited and inconclusive. Accordingly, this cross-sectional study aimed to comprehensively examine the interrelationships among physical activity, perceived stress, and oral health in university students, in order to provide empirical evidence to inform targeted oral health promotion strategies in this population. The null hypothesis was that there are no significant associations among physical activity, perceived stress, and oral health in university students.

## 2. Methods

### 2.1. Study design

From April 12 to May 12, 2026, a cross-sectional survey was conducted among university students enrolled in mainland China. This investigation was conducted in accordance with the Declaration of Helsinki and was approved by the institutional ethics committee (approval No. [2026]-SR-52). Informed consent was obtained from all subjects involved in the study. Data were collected using an online self-administered questionnaire hosted on Questionnaire Star, a widely used online survey platform in China. The online questionnaire link was distributed via posters released by university student unions and student WeChat social groups across several universities in mainland China. Eligibility criteria were: age ≥ 18 years and current enrollment as a university student. Prior to enrollment, all potential participants were informed in detail about the study purpose, inclusion criteria, procedures for ensuring data confidentiality, and their unrestricted right to discontinue participation at any stage. Access to the questionnaire was restricted to individuals who explicitly agreed to participate by providing informed consent.

### 2.2. Measurements

#### 2.2.1. Demographic characteristics

Demographic information collected from participants included age, gender, height, weight, only-child status, educational level, and parental educational attainment. Smoking and alcohol consumption were categorized as current, never, or former, and Sleep length was divided into 3 distinct groups: >8, 6 to 8, and < 6 hours. The calculation of body mass index relied on self-reported height and weight data.

#### 2.2.2. International Physical Activity Questionnaire Short Form

Participants’ physical activity status was evaluated via the International Physical Activity Questionnaire Short Form (IPAQ-SF). This 7-item scale collects data on weekly physical activity duration and daily sedentary time.^[16]^ The questionnaire classifies movements into 3 intensity tiers according to metabolic equivalent (MET) values: vigorous activity at 8.0 METs, moderate activity at 4.0 METs, and walking or light activity at 3.3 METs. Total weekly physical activity was computed using the formula: Total MET-min/week = (walking METs × minutes × days) + (moderate METs × minutes × days) + (vigorous METs × minutes × days). In line with the official scoring criteria of IPAQ-SF, respondents reaching 600 or more MET-min per week were defined as having adequate physical activity, while those below this threshold were considered physically inactive.^[7]^ Prior research has verified the reliability and construct validity of the Chinese adapted version of IPAQ-SF.^[17]^

#### 2.2.3. Perceived Stress Scale 14

Stress was assessed using the 14-item Perceived Stress Scale (PSS-14),^[18]^ which measures the degree of stress experienced over the preceding month in response to various life situations. Each question is assessed via a 5-point Likert scale, where 0 represents “never” and 4 represents “very often.” This scale generates a total score between 0 and 56. Respondents were classified into 3 stress categories based on their cumulative PSS-14 scores: 0 to 28, normal stress; 29 to 42, high stress; and > 42, excessive stress. Previous studies have reported satisfactory internal consistency for the PSS-14,^[19]^ and the Chinese version has been validated.^[20]^

#### 2.2.4. Oral health status

Oral health status was assessed using multiple self-reported indicators, including the presence of toothache, gingival bleeding, and oral ulceration. Participants were asked to report whether they had experienced toothache, gingival bleeding, or oral ulcers (0 = no, 1 = yes). In addition, oral health-related behaviors, specifically toothbrushing frequency and dental floss use, were assessed.

### 2.3. Statistical analysis

All statistical procedures were carried out with SPSS 27.0 (IBM). Missing data were handled using listwise deletion. Participants with missing values in analytical variables, including demographics, physical activity, perceived stress, and oral‑health‑related indicators, were excluded from the analytical dataset. No imputation methods were applied in the present study. Categorical data were described by counts and proportional values, while continuous data were presented as mean values alongside standard deviations. The Kolmogorov–Smirnov test was applied to verify data normality, and Levene test was adopted to check the homogeneity of variance across groups. Chi-square tests were utilized to compare oral health conditions for categorical indicators, and independent *t* tests were employed for continuous indicators. Multivariate logistic regression analyses were established to explore the relationships between stress levels, physical activity, oral health behaviors, and oral health outcomes. Potential confounding factors including age, body mass index, gender, only-child status, parental education, participants’ educational background, smoking and drinking habits as well as sleep duration were controlled in all regression models. The outcomes were reported as odds ratios (OR) and their corresponding 95% confidence intervals (CI).

Structural equation modeling (SEM) was further performed via Mplus 8.3 software to systematically clarify the intricate relationships among physical activity, stress, and oral health. In the present model, self-perceived oral symptoms were defined as a latent construct reflected by 3 measurable indicators, namely toothache, gingival bleeding, and oral ulcers. Unlike observable variables, this latent oral symptom construct cannot be directly quantified and was therefore estimated based on the corresponding 3 observed behavioral and symptomatic indices in the established SEM framework. To validate the structural equation model developed in the current research, a set of commonly accepted goodness-of-fit statistics were utilized. These evaluative measures included the Root Mean Square Error of Approximation (RMSEA), Tucker–Lewis Index (TLI), Comparative Fit Index (CFI), and Standardized Root Mean Square Residual (SRMR), which are universally applied to judge the overall compatibility between the empirical data and the hypothetical model. In accordance with the well-established model fit evaluation criteria, a model was regarded as having acceptable fitting performance when the RMSEA value was lower than 0.1, CFI and TLI values exceeded 0.90, and the SRMR value was <0.08.^[21]^ A *P* value < .05 was considered statistically significant.

## 3. Results

Of the 1676 initially collected survey responses, 98 incomplete questionnaires were excluded, and data from 1578 participants were included in the final analysis. Table 1 summarizes data from 1578 participants with a mean age of 22.68 years (standard deviation = 2.46), of whom 35.1% were male and 64.9% were female. Among all participants, 61.2% reported normal stress, 36.2% high stress, and 2.5% excessive stress. Physical activity was insufficient in 47.7% of the sample and sufficient in 52.3%. Several factors showed statistically significant associations with the 3 oral health conditions. Toothache was significantly associated with sleeping <6 hours, high or excessive stress, brushing less than twice daily, and flossing less than once daily. Gingival bleeding was significantly associated with smoking status, sleeping <6 hours, high stress, insufficient physical activity, infrequent toothbrushing, and infrequent flossing. Oral ulcers were significantly associated with high stress and insufficient physical activity. In contrast, no statistically significant associations with any of the 3 conditions were observed for age, gender, overweight status, only-child status, education level, parental education, or alcohol consumption.

**Table 1:** Sample characteristics.

Characteristics	Category	Total	Toothache			Gingival bleeding			Oral ulcer		
			Yes	No	*P*	Yes	No	*P*	Yes	No	*P*
Age	Mean (SD)	22.68 (2.46)	22.46 (2.44)	22.75 (2.46)	.93	22.49 (2.36)	22.78 (2.50)	.23	22.61 (2.45)	22.46 (2.46)	.67
Gender	Male	554 (35.1%)	127 (33.2%)	427 (35.7%)	.36	181 (34.5%)	373 (35.4%)	.71	128 (37.5%)	336 (33.7%)	.13
	Female	1024 (64.9%)	256 (66.8%)	768 (64.3%)		344 (65.5%)	680 (64.6%)		363 (62.5%)	661 (66.3%)	
Overweight	Yes	270 (17.1%)	63 (16.4%)	207 (17.3%)	.69	80 (15.2%)	190 (18.0%)	.16	98 (16.9%)	172 (17.3%)	.85
	No	1308 (82.9%)	320 (83.6%)	988 (82.7%)		445 (84.8%)	863 (82.0%)		483 (83.1%)	825 (82.7%)	
Only one-child	Yes	755 (47.8%)	184 (48.0%)	571 (47.8%)	.93	254 (48.4%)	501 (47.6%)	.76	276 (47.5%)	479 (48.0%)	.84
	No	823 (52.2%)	199 (52.0%)	624 (52.2%)		271 (51.6%)	552 (52.4%)		305 (52.5%)	518 (52.0%)	
Education	Undergraduate	853 (54.1%)	209 (54.6%)	644 (53.9%)	.43	293 (55.8%)	560 (53.2%)	.51	313 (53.9%)	540 (54.1%)	.82
	Master	606 (38.4%)	140 (36.6%)	466 (39.9%)		191 (36.4%)	415 (39.4%)		221 (38.0%)	385 (38.6%)	
	Doctor	119 (7.5%)	34 (8.9%)	85 (7.1%)		41 (7.8%)	78 (7.4%)		47 (8.1%)	72 (7.2%)	
Parental education level	>High school	643 (40.7%)	145 (37.9%)	498 (41.7%)	.19	205 (39.0%)	438 (41.6%)	.33	223 (38.4%)	420 (42.1%)	.14
	≤High school	935 (59.3%)	238 (62.1%)	697 (58.3%)		320 (61.0%)	615 (58.4%)		358 (61.6%)	577 (57.9%)	
Smoking	Current	128 (8.1%)	27 (7.0%)	101 (8.5%)	.26	30 (5.7%)	98 (9.3%)	.01*	52 (9.0%)	76 (7.6%)	.58
	Never	1396 (88.5%)	347 (90.6%)	1049 (87.8%)		482 (91.8%)	914 (86.8%)		511 (88.0%)	885 (88.8%)	
	Previous	54 (3.4%)	9 (2.3%)	45 (3.8%)		13 (2.5%)	41 (3.9%)		18 (3.1%)	36 (3.6%)	
Drinking	Current	652 (41.3%)	162 (42.3%)	490 (41.0%)	.88	212 (40.4%)	440 (41.8%)	.85	238 (41.0%)	414 (41.5%)	.83
	Never	877 (55.6%)	210 (54.8)	667 (55.8%)		297 (56.6%)	580 (55.1%)		323 (55.6%)	554 (55.6%)	
	Previous	49 (3.1%)	11 (2.9%)	38 (3.2%)		16 (3.0%)	33 (3.1%)		20 (3.4%)	29 (2.9%)	
Sleeping	<6h	137 (8.7%)	54 (14.1%)	83 (6.9%)	<.01*	61 (11.6%)	76 (7.2%)	.01*	62 (10.7%)	75 (7.5%)	.10
	6–8h	1071 (67.9%)	241 (62.9%)	830 (69.5%)		351 (66.9%)	720 (68.4%)		386 (66.4%)	685 (68.7%)	
	>8h	370 (23.4%)	88 (23.0%)	282 (23.6%)		113 (21.5%)	257 (24.4%)		133 (22.9%)	237 (23.8%)	
Stress	Normal	966 (61.2%)	201 (52.5%)	765 (64.0%)	<.01*	288 (54.9%)	678 (64.4%)	<.01*	338 (58.2%)	628 (63.0%)	.01*
	High	572 (36.2%)	169 (44.1%)	403 (33.7%)		217 (41.3%)	335 (33.7%)		220 (37.9%)	352 (35.3%)	
	Excessive	40 (2.5%)	13 (3.4%)	27 (2.3%)		20 (3.8%)	20 (1.9%)		23 (4.0%)	17 (1.7%)	
Physical activity	Insufficient	752 (47.7%)	181 (47.3%)	571 (47.8%)	.86	271 (51.6%)	481 (45.7%)	.03*	299 (52.5%)	453 (45.4%)	.02*
	Sufficient	826 (52.3%)	202 (52.7%)	624 (52.2%)		254 (48.4%)	572 (54.3%)		282 (48.5%)	544 (54.6%)	
Toothbrushing	≥2 times a day	1159 (73.4%)	265 (69.2%)	894 (74.8%)	.03*	369 (70.3%)	790 (75.0%)	.04*	411 (70.7%)	748 (75.0%)	.06
	<2 times a day	419 (26.6%)	118 (30.8%)	301 (25.2%)		156 (29.7%)	263 (25.0%)		170 (29.3%)	249 (25.0%)	
Dental floss	≥once a day	357 (22.6%)	62 (16.2%)	295 (24.7%)	<.01*	102 (19.4%)	255 (24.2%)	.03*	120 (20.7%)	237 (23.8%)	.15
	<once a day	1221 (77.4%)	321 (83.8%)	900 (75.3%)		423 (80.6%)	798 (75.8%)		461 (79.3%)	760 (76.2%)	

* *P* < .05 indicates statistically significant differences between groups for the respective oral health condition (based on chi‑square test for categorical variables or independent-samples t test for continuous variables).

Table 2 presents the multivariable logistic regression analysis of factors associated with oral health conditions. Stress level and physical activity were examined in relation to 3 outcomes, using “excessive” stress and “sufficient” physical activity as the reference categories. For gingival bleeding, participants with normal stress had a significantly lower risk (OR = 0.50, 95% CI: 0.26–0.97, *P* = .04), whereas insufficient physical activity was associated with an increased risk (OR = 1.29, 95% CI: 1.04–1.60, *P* = .02). For oral ulcers, both normal stress (OR = 0.44, 95% CI: 0.23–0.85, *P* = .02) and high stress (OR = 0.51, 95% CI: 0.26–0.98, *P* = .04) were associated with significantly lower odds compared with excessive stress, while insufficient physical activity was again a significant risk factor (OR = 1.25, 95% CI: 1.02–1.55, *P* = .04). For toothache, no statistically significant associations were observed: normal stress (OR = 0.77, *P* = .47), high stress (OR = 1.19, *P* = .63), and insufficient physical activity (OR = 0.95, *P* = .70).

**Table 2:** Multivariate logistic regression of factors associated with oral health condition.

Characteristics	Category	Toothache			Gingival bleeding			Oral ulcer		
		Wald Χ^2^	*P*	OR (95% CI)	Wald Χ^2^	*P*	OR (95% CI)	Wald Χ^2^	*P*	OR (95% CI)
Age	Mean (SD)	5.47	.02	0.92 (0.86–0.99)	3.23	.07	0.95 (0.89–1.01)	1.32	.25	0.97 (0.91–1.03)
Gender	Male	1.47	.23	0.85 (0.66–1.11)	0.19	.67	0.95 (0.75–1.20)	1.86	.17	1.17 (0.93–1.47)
	Female			Ref			Ref			Ref
Overweight	No	0.12	.91	0.98 (0.71–1.35)	0.57	.45	1.19 (0.83–1.50)	0.01	.96	1.01 (0.76–1.33)
	Yes			Ref			Ref			Ref
Only one-child	Yes	0.03	.86	1.02 (0.81–1.30)	0.54	.46	1.09 (0.87–1.35)	0.01	.94	1.01 (0.82–1.24)
	No			Ref			Ref			Ref
Education	Undergraduate	5.64	.02	0.52 (0.30–0.89)	1.17	.28	0.76 (0.46–1.26)	1.12	.29	0.77 (0.47–1.25)
	Master	3.30	.07	0.65 (0.41–1.04)	0.85	.36	0.81 (0.53–1.26)	0.70	.40	0.83 (0.55–1.28)
	Doctor			Ref			Ref			Ref
Parental education level	>High school	1.42	.23	0.86 (0.68–1.10)	0.54	.46	0.92 (0.74–1.15)	2.29	.13	0.85 (0.69–1.05)
	≤High school			Ref			Ref			Ref
Smoking	Current	0.46	.50	1.35 (0.57–3.20)	0.02	.88	0.94 (0.44–2.03)	0.86	.35	1.39 (0.70–2.75)
	Never	2.20	.14	1.78 (0.83–3.83)	2.63	.11	1.73 (0.89–3.36)	0.29	.59	1.18 (0.65–2.15)
	Previous			Ref			Ref			Ref
Drinking	Current	0.02	.88	1.06 (0.51–2.18)	0.05	.83	0.93 (0.49–1.78)	0.35	.55	0.83 (0.45–1.53)
	Never	0.07	.79	0.91 (0.44–1.88)	0.21	.65	0.86 (0.45–1.64)	0.34	.56	0.83 (0.45–1.54)
	Previous			Ref			Ref			Ref
Sleeping	<6 h	9.22	<.01	1.96 (1.27–3.02)	5.35	.02	1.63 (1.08–2.47)	2.16	.14	1.36 (0.90–2.04)
	6–8 h	0.88	.35	0.87 (0.66–1.16)	0.27	.61	1.07 (0.83–1.39)	0.01	.95	0.99 (0.77–1.27)
	>8 h			Ref			Ref			Ref
Stress	Normal	0.53	.47	0.77 (0.38–1.56)	4.26	.04	0.50 (0.26–0.97)	5.92	.02	0.44 (0.23–0.85)
	High	0.24	.63	1.19 (0.48–2.44)	1.06	.30	0.71 (0.36–1.37)	4.08	.04	0.51 (0.26–0.98)
	Super			Ref			Ref			Ref
Physical activity	Insufficient	0.15	.70	0.95 (0.75–1.21)	5.31	.02	1.29 (1.04–1.60)	4.47	.04	1.25 (1.02–1.55)
	Sufficient			Ref			Ref			Ref
Toothbrushing	≥2 times a day	2.87	.09	0.79 (0.61–1.04)	2.17	.14	0.83 (0.65–1.06)	1.47	.23	0.86 (0.68–1.10)
	<2 times a day			Ref			Ref			Ref
Dental floss	≥once a day	10.31	<.01	0.60 (0.44–0.82)	2.78	.10	0.80 (0.61–1.04)	0.63	.43	0.90 (0.70–1.17)
	<once a day			Ref			Ref			Ref

**Figure 1.**
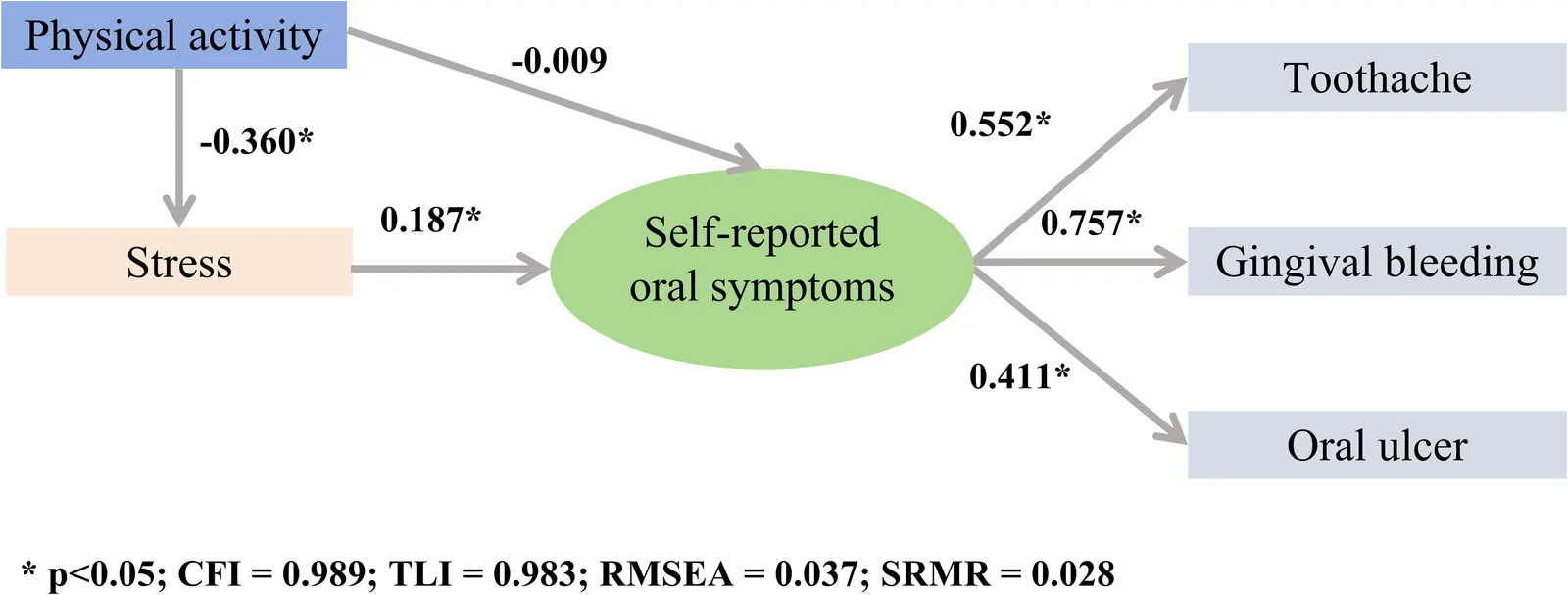
Standardized coefficients for the structural model testing the mediation effect of stress on the relationship between physical activity and self-reported oral symptoms. CFI = Comparative Fit Index, RMSEA = Root Mean Square Error of Approximation, SRMR = Standardized Root Mean Square Residual, TLI = Tucker‑Lewis Index.

Figure 1 presents the standardized path coefficients from the SEM, which demonstrated good fit to the data (CFI = 0.989; TLI = 0.983; RMSEA = 0.037; SRMR = 0.028). Physical activity showed a significant negative association with stress (standardized coefficient = −0.360, *P* < .05), whereas stress was significantly positively associated with self‑reported oral symptoms (standardized coefficient = 0.411, *P* < .05). For the 3 observed indicators of the latent construct of self‑perceived oral symptoms, all demonstrated significant positive factor loading: toothache (standardized coefficient = 0.187, *P* < .05), gingival bleeding (standardized coefficient = 0.552, *P* < .05), and oral ulcer (standardized coefficient = 0.757, *P* < .05), with oral ulcer showing the strongest loading on the latent oral symptom factor. The direct path from physical activity to the latent self‑reported oral symptom construct was non‑significant (standardized coefficient = −0.009, *P* = .765).

## 4. Discussion

The present investigation explored the patterns of physical activity, perceived stress, and oral health status, as well as their interrelationships, among Chinese university students. Our findings revealed a relatively low proportion of students meeting recommended levels of physical activity (52.3%) and a comparatively high prevalence of elevated stress (38.8%). Insufficient physical activity and higher stress were all associated with self‑reported gingival bleeding and oral ulcer, whereas toothache did not show a direct relationship with physical activity or stress in this population. To our knowledge, this is the first investigation to assess the potential links between physical activity, perceived stress, and oral health status among university students in China.

Physical activity is widely endorsed by leading medical and public health organizations as a key population-level strategy for preventing numerous psychiatric and somatic disorders.^[22]^ As a health-promoting behavior, it is recommended for both healthy individuals and those with existing conditions to improve overall quality of life. Regular physical activity has also been positively related to higher academic performance.^[23]^ Consequently, adherence to guideline-based levels of physical activity is particularly important for students’ holistic development. In the present study, however, only 52.3% students met the criteria for sufficient physical activity. This finding highlights the need for targeted measures to increase physical activity as a means of promoting both oral and psychological well-being. Previous research has identified social support as an important determinant of physical activity in this population.^[24]^ Establishing student clubs or interest groups focused on specific physical activities may foster a socially supportive and motivating context for regular exercise. In addition, technology-based approaches, such as smartphone applications and wearable activity trackers, have demonstrated efficacy in increasing physical activity among young adults. Direito et al reported that mobile app-based interventions can significantly increase activity levels by providing tailored feedback, goal-setting tools, and progress-monitoring features.^[25]^ Integrating social support initiatives with digital interventions may therefore represent a synergistic strategy for cultivating and maintaining higher physical activity levels in Chinese university settings.

Stress is an inevitable component of daily life and exerts substantial effects on physical health. Owing to constrained free time, prolonged instructional hours, frequent examinations, and heavy academic workloads, university students experience higher stress levels than the general population.^[26–28]^ In this study, PSS‑14 scores indicated that perceived stress was markedly elevated in this group. Among all participants, 36.2% of respondents reported high stress, and 2.5% excessive stress. This high prevalence of psychological distress likely serves as a critical determinant of oral health through both behavioral and physiological pathways. Behaviorally, students facing intense academic pressure may neglect routine self-care practices, such as consistent toothbrushing and flossing, which further exacerbate oral conditions.^[29]^ Physiologically, chronic stress may lead to improved cortisol levels that can suppress immune function and heighten systemic inflammation.^[30]^ Consequently, oral health promotion within university settings should not be viewed in isolation; instead, it must be integrated with mental health support and stress-management interventions. Providing students with effective coping strategies and promoting a balanced lifestyle are essential steps to mitigate the detrimental impact of academic pressure on both their psychological well-being and their long-term oral health outcomes.

Multivariable logistic regression analyses revealed significant associations between physical activity and both gingival bleeding and oral ulceration, consistent with previous findings.^[31]^ Although logistic regression identified these bivariate‑adjusted associations, our subsequent SEM further elucidated the underlying mechanistic pathways and enabled us to distinguish direct versus mediated effects of physical activity on oral‑health outcomes. The SEM results confirmed that perceived stress functions as a key mediator: physical activity was significantly and negatively associated with stress, which in turn was a strong positive predictor of oral symptoms. Notably, the direct path from physical activity to the latent oral‑symptom construct was non‑significant This indicates that, in the present sample of university students, the oral‑health‑related benefits of physical activity operate primarily through stress reduction rather than via a direct independent effect.

Biologically, previous studies have shown that regular exercise can influence circulating C‑reactive protein, a marker closely associated with periodontitis and partly related to vitamin K metabolism.^[32]^ The beneficial effects of physical activity on periodontal status are thought to stem from its systemic anti‑inflammatory properties and its ability to improve endothelial function.^[33]^ It is worth noting that such systemic anti‑inflammatory effects of physical activity may still exist in this population; however, these effects were not reflected as a statistically significant direct association with self‑reported oral symptoms in our mediation model. By lowering perceived stress, physical activity may further prevent stress‑induced immune suppression and the worsening of oral inflammatory conditions such as gingival bleeding and oral ulcers. The discrepancy between the significant findings observed in multivariable logistic regression and the non‑significant direct SEM path may partly arise because logistic regression does not partition total effects into direct and indirect components. Logistic regression captures the total association between physical activity and oral symptoms without accounting for the mediating role of perceived stress, whereas SEM explicitly separates these components. Future longitudinal studies with clinical oral examinations will be valuable to further disentangle potential direct anti‑inflammatory influences of physical activity from stress‑driven indirect pathways.

This study has several limitations. First, its cross-sectional design prevents the establishment of definitive causal relationships between physical activity, stress, and oral health, as the data only capture a single point in time. Second, the reliance on self-reported measures for oral symptoms may introduce recall or social desirability bias, which might differ from results obtained through objective clinical dental examinations. Third, the recruitment approach may introduce selection bias. Students who notice student‑union posters or frequently participate in student WeChat social groups are more likely to complete our survey, which could limit the generalizability of findings. Consequently, future research should employ longitudinal designs and more randomized sampling methods to better understand the long-term developmental trajectories of these associations.

## 5. Conclusion

This study underscores the critical interrelationships between physical activity, perceived stress, and oral health among university students, revealing that insufficient physical activity and elevated stress levels are significantly associated with a higher prevalence of oral symptoms, particularly gingival bleeding and oral ulcers. Physical activity is negatively associated with stress, which in turn acts as a significant predictor of self-reported oral health issues. Accordingly, there is an urgent need to implement integrated interventions to encourage physical activity and stress management, ultimately enhancing the holistic well-being and oral health of the student population. Future longitudinal studies are warranted to further elucidate the causal mechanisms between physical activity and oral health, while incorporating objective clinical evaluations to validate these self-reported findings across more diverse populations.

## Acknowledgments

The authors sincerely thank all participants for their valuable contributions to this study.

## Author contributions

**Writing – original draft:** Qianru Li, Zhe Chen, Chenyuan Sun, Shengyun Lin.

**Writing – review & editing:** Xinhan Yang, Jian Li.
